# MicroRNA-26b inhibits cell proliferation and cytokine secretion in human RASF cells via the Wnt/GSK-3β/β-catenin pathway

**DOI:** 10.1186/s13000-015-0309-x

**Published:** 2015-06-19

**Authors:** Jiling Sun, Peng Yan, Yuanzheng Chen, Yang Chen, Jianxun Yang, Guangyue Xu, Haijun Mao, Yong Qiu

**Affiliations:** Nursing Office, Linyi People’s Hospital, Linyi, 276000 P. R. China; Department of Rheumatology, Linyi People’s Hospital, Linyi, 276000 P. R. China; Department of Burn and Plastic Surgery, Linyi People’s Hospital, Linyi, 276000 P. R. China; Department of Orthopedics, Linyi People’s Hospital, North of Yimeng Road, Lanshan District, Linyi, 276000 P. R. China; Department of Orthopaedics, Nanjing Drum Tower Hospital The Affiliated Hospital of Nanjing University Medical School, Nanjing, 210008 P. R. China

**Keywords:** MicroRNA-26b, Rheumatoid arthritis, Wnt/GSK-3β/β-catenin pathway, CyclinD1, Cytokine

## Abstract

**Background:**

Rheumatoid arthritis (RA) is a chronic systemic auto- immune disease characterized by joint synovitis. Recent evidence suggests that rheumatoid arthritis synovial fibroblasts (RASFs) promote joint destruction. In this study, we investigated the role of microRNA-26b (miR-26b) in cell proliferation and inflammatory cytokine secretion using patient-derived Rheumatoid arthritis fibroblast-like synoviocyte (RAFLS) to understand pathways influencing rheumatoid arthritis.

**Methods:**

RAFLS were cultured in vitro and transfected with miR-26b mimics (experimental group) and negative sequence (control group). The protein levels of Wnt4, Wnt5ɑ, GSK-3β, CyclinD1, Ser9-GSK-3β and β-catenin were detected by western blot analysis. Tumor Necrosis Factor-ɑ (TNF-ɑ), IL- 1β, and IL-6 levels were quantified by Enzyme-linked Immunosorbent Assay (ELISA). RAFLS proliferation and apoptosis were measured by 3-[4, 5-dimethylthiazol-2-yl]-2, 5-diphenyl tetrazolium bromide (MTT) assay and flow cytometry, respectively.

**Results:**

GSK-3β and CyclinD1 expression levels were lower in miR-26b mimic group compared to Mock group and negative control (NC) group. Conversely, GSK-3β and CyclinD1 expression levels were markedly higher in the miR-26b inhibitor group compared to Mock and NC group (*P* < 0.05). Transfection of miR-26b mimics significantly increased the, levels of Ser9-GSK-3β and β-catenin in comparison to Mock and NC groups, while transfection of miR-26b inhibitors showed the opposite effect. In miR-26b mimic group, TNF-α, IL- 1β and IL-6 levels were lower than the Mock and NC groups, while in miR-26b inhibitor group, these cytokine levels were higher than the Mock and NC groups (*P* < 0.05). Transfection of miR-26b mimics significantly reduced the cell proliferation of RAFLS, compared to the Mock and NC groups, and miR-26b inhibitors increased the proliferative capacity of RAFLS compared to Mock and NC groups (*P* < 0.05). The miR-26b mimic group exhibited higher RAFLS apoptosis rate compared to Mock and NC group and miR-26b inhibitor group showed significantly lower RAFLS apoptosis rate compared to Mock and NC groups (*P* < 0.05).

**Conclusions:**

MiR-26b regulates β-catenin and CyclinD1 levels by inhibiting GSK-3β expression, which in-turn alters the Wnt/GSK-3β/β-catenin pathway to lower RAFLS proliferation and elevate cell apoptosis and the secretion of TNF-α,IL-1β and IL-6 cytokines. Therefore, our results show that miR-26B plays a central role in inhibiting the inflammation associated with rheumatoid arthritis.

**Virtual Slides:**

The virtual slide(s) for this article can be found here: http://www.diagnosticpathology.diagnomx.eu/vs/9063056861547150

## Background

Rheumatoid arthritis (RA) is a chronic systemic autoimmune disease characterized by joint synovitis [1, 2]. The clinical manifestations of RA includes joint swelling and pain caused by synovitis, cartilage destruction, joint space narrowing, joint stiffness, deformity and dysfunction, which are directly related to primary chronic low-grade inflammation [3, 4]. RA affects 0.5-1 % of adults in developed countries and approximately 5–50 per 100,000 population in developing countries each year [5]. RA onset is rare under the age of 15, but its incidence shows a steady increase with age until 80, with women 3–5 times more susceptible than men [6]. The exact cause of RA is still unknown, but genetic factors, such as human leukocyte antigen-DR4 (*HLA-DR4*) and other non-HLA genes including protein tyrosine phosphatase, non-receptor type 22 (*PTPN22*) and peptidyl arginine deiminase, type IV (*PADI4*), are suspected as major contributing factors [7, 8]. Non-genetic factors also contribute significantly to RA and include Epstein-Barr virus (EBV) and Human Herpes Virus 6 (HHV-6) infections, hormonal infleunces, smoking, cold temperatures and trauma [9, 10]. Previous studies show that loss of balance in proliferation and apoptosis of synovial fibroblast (SF) and abnormal secretion of various cytokines play key roles in RA pathogenesis. Multiple signaling pathways are activated during RA development [11, 12]. Synovial tissue from RA patients shows infiltration by macrophages, T cells, and B cells, proliferation of cells lining the synovium, and production of inflammatory cytokines such as tumor necrosis factor α (TNFα) and interleukin-1β (IL-1β) [13, 14]. Interestingly, inhibition of these cytokines ameliorates the clinical symptoms RA, strongly supporting the central role of cytokines in RA [15]. Rheumatoid arthritis synovial fibroblast (RASFs) activity promotes joint destruction and increased expression of proinflammatory pathways and secretion of matrix-destructive enzymes is a common feature associated with the disease [16]. Recent evidence suggests that miRNA dysregulation may contribute to RA etiopathogenesis and therefore, a better understanding of pathways regulated by miRNAs might shed light on RA pathogenesis and help identify effective RA treatments [17].

MicroRNAs (miRNAs) are small, non-coding endogenous RNAs of 20 ~ 24 nucleotides in length and regulate gene expressions at the post-transcriptional level [18]. MiRNAs bind to 3′ untranslated regions (3′ UTRs) of their target mRNAs and either block translation and/or promote target mRNA degradation [19]. MiRNAs play important roles both in pathological and normal physiological processes such as embryonic development, energy homeostasis, metabolism of sugar and lipid as well as tumorigenesis [20–23]. Several miRNAs modify cell behavior by regulating the nuclear factor-kB (NF-κB) pathway [18]. For instance, miR-30e, miR-182, and miR-301a promote NF-κB activity to enhance tumor growth, invasiveness or angiogenesis [24–26]. Joanna Stancz et al. observed dysregulated expression of miRNA miR-155 and miR-146a in synovial tissue, synovial fibroblasts and monocytes of rheumatoid joints [16]. Previous studies showed that the miR-26 family, consisting of miR-26a and miR-26b, is down-regulated in several cancers such as hepatocellular carcinoma (HCC), melanoma, nasopharyngeal carcinoma and breast cancer [27–31]. Although the cellular functions of miR-26b remain elusive, miR-26b inhibits NF-κB pathway in HCC cells by suppressing TAK1 and TAB3 expression, and down-regulation of miR-26b suppressed apoptosis in HCC cells [18]. Thus far, the main role of miR-26b was described in cancers. However, due to the close relationship between cancer and inflammatory pathway, we investigated whether miR-26b influenced cell proliferation and inflammatory cytokine secretion in RASF cells and further sought to identify the underlying mechanisms.

## Methods

### Ethics Statement

The study was approved by the Institutional Review Boards (IRBs) of Linyi People’s Hospital. Written informed consent was obtained from each eligible participant and the study was performed in accordance with the Declaration of Helsinki.

### Culture of rheumatoid arthritis fibroblast-like synoviocytes (RAFLS)

Human synovial tissues from the affected joints were collected from patients admitted at the Linyi People’s Hospital signed informed consent forms were obtained before the procedure. The diagnosis of RA in these patients was according to American Rheumatism Association 1987 revised criteria for classification of rheumatoid arthritis [32]. Synovial tissues were obtained from RA patients at surgery. They collected tissue samples were minced and digested with 2.5 g/L trypsase for 2 h at 37 °C and cells were collected after centrifugation and cultured in Dulbecco’s minimum essential medium (DMEM) in 5 % CO_2_ at 37 °C. The cells were cryopreserved between 3^rd^ – 8^th^ passage and cells within these passages were chosen for all experiments.

### Cell grouping and transfection

The experimental set-up consisted of 4 groups: the Mock group, negative control (NC group transfected with miR-26b negative control sequence), miR-26b mimics group (transfected with miR-26b mimics) and the miR-26b inhibitor group (transfected with miR-26b inhibitor). RAFLS in logarithmic phase cultured in DMEM with 10 % FBS were detached to for single cell suspension. They were adjusted to 1 × 10^6^ cell/ml concentration and seeded into 6-well culture plates. Cell transfections were performed when the seeded cells reached 80 % confluency. Lipofectamine 2000 (Invitrogen Company, USA) was used to transfect the cells. Plasmids (1 μg) carrying the respective miR-26b sequences were mixed separately with serum-free medium and 2.5 μl lipofectamine 2000 and incubated at room temperature for 15 min under serum-free conditions to form transfection complexes. The RAFLS were washed twice with PBS and the corresponding transfection complexes were added to each well. Complete DMEM media was replaced 6 h later. The transfection efficiency was verified by fluroscence microscopy at 24 h and 48 h, and cells at 48 h post-transfection were used for further analsis.

### Real-time quantitative polymerase chain reaction (PCR) technique for measurement of miR-26b expression

Trizol reagent was used to purify total RNA and 0.05 μg of the total RNA was used for first strand cDNA synthesis using SuperscriptII reverse transcriptase. Specific primers for miRNA for real-time quantitative PCR and first strand cDNA synthesis were based on published sequences. Light Cycler PCR and the reagents were lightcycler-faststart DNA master SYBR green were from Roche. Reaction conditions were: magnesium concentration was 3 mmol/L, primer concentrations were 0.25 μmol/L; denaturation for15 s at 95 °C, 60 °C and then anneal for 30 s, repeated 40 cycles. Solubility curve were protracted at 70 °C to 95 °C and PCR products were verified by electrophoresis.

### Dual-luciferase enzyme assay system for reporter gene analysis

The cells were transfected as mentioned above and the transfected cells at 48 h were washed with 1 × PBS. The cells were treated with lysis buffer and Dual-Luciferase® Reporter Assay (Promega) was performed, using luminometer, to measure the activity of luciferase. The firefly luciferase activity values were recorded and compared to the Ranilla luciferase activity to quantify transfection efficiency.

### Western blot

Wnt4, Wnt5α, GSK-3β, CyclinD1, Ser9-GSK-3β and β-catenin protein levels were detected by western blot analysis. RIPA lysis buffer was used to exact proteins and BCA method was used to estimate total protein concentration. The samples were electrophoresed in 10 % SDS-PAGE gels (100v, 100 min). The proteins were transferred to PVDF membranes (Invitrogen company) and blocked with 10 % skimmed milk. Next, corresponding primary antibodies (1: 1000) were used to detected the protein, the membranes were washed with TBST thrice and incubated with horseradish peroxidase (HRP)-labeled secondary antibody for 1 h and DAB staining was performed after washing the membranes, followed by recording the results using gel imaging system. The quantitative measurement of the proteins was as follows: relative expression of the target protein = grey level of target band/grey level of the same sample reference.

### Experimental Study of Enzyme-linked Immunosorbent Assay (ELISA) method

Tumor Necrosis Factor-α (TNF-α), IL- 1β, and IL-6 levels were detected using ELISA kit (BD Company, USA). The cells were centrifuged at 1500 rpm for 5 min in 4 °C. The samples were resuspended at 1 × 10^6^ cells/ml and seeded into 12-well plates. Criss-cross serial dilution analysis was adopted to ensure the cell numbers and concentration of labelled complex. From each well, 100 μl cell suspensions were added to ELISA plate, centrifuged at 1500 rpm for 1 min, incubated to allow coating and then the supernatants were removed. To each well, 100 μl of antibody solution diluted in PBS was used and incubated for 90 min at 4 °C. Next, 100 μl enzyme-antibody complex diluted in PBS was added to each well and incubated for 90 min at 4 °C and washed 3 times. A 100 μl volume of substrate solution was added to each well and incubated for 1 h at room temperature and the detection was at 450 nm to measure the absorbance.

### Cell proliferation detected by 3-[4, 5-dimethylthiazol-2-yl]-2, 5-diphenyl tetrazolium bromide (MTT) assay

RAFLS in each group were collected and the cell concentration was adjusted to 1 × 10^6^ cells/ml, and inoculated into 96-well culture plate (100 μL/well) for various times. The cells were incubated with 20 μL MTT (5 mg/ml, Sigma Chemicals Co.) for 4 h. Next, 200 μl DMSO were added to each well and incubated at room temperature for 10 min in dark to dissolve the complexes. To plot the MTT curves, absorbance values were used as ordinate and time interval was the abscissa. All samples were in triplicates and the experiment was repeated 3 times.

### Cell apoptosis assayed by flow cytometry

AnnexinV-FITC (Sigma Chemicals Co) was used according to manufacturer’s instructions. Briefly, cells from each group were washed 3 times in pre-cooled PBS. A volume of 1 μl 1 × annexin V was combined with buffer solution, centrifuged to discard the liquid supernatant and 200 μL binding buffer added to resuspend the cells. A volume of 10 μL annexin V-FITC and 5 μl PI (5 mg/L) were added, mixed and incubated for 30 min in dark, followed by flow cytometry (Olympus company) to quantify the apoptosis rate.

### Statistical analysis

All statistical analyses were performed using SPSS 18.0 software (SPSS, Inc., Chicago, IL, USA). All data were expressed as mean ± SEM. Differences between groups were measured using independent samples *t*-test. A *P* < 0.05 value denoted statistically significant difference.

## Results

### Expression of green fluorescent protein (GFP) after RAFLS transfection in each group

**Fig. 1:**
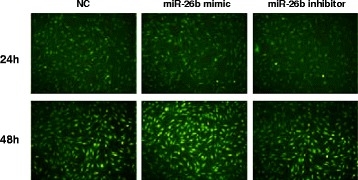
Expression of green fluorescent protein (GFP) in RAFLS at 24 and 48 h post-transfection

Following the transfection of RAFLS with various plasmids as indicated, the GFP marker expressed by the recombinant plasmid was used to determine transfection efficiency by fluorescence microscopy (Fig. 1). After 24 h of transfection, GFP express was visible and this expression significantly increased after 48 h.

### Confirmation of miR-26b expression by real-time quantitative PCR

**Fig. 2:**
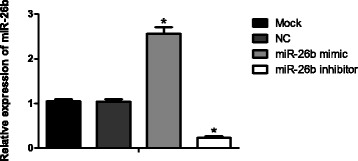
The expression of miR-26b in each transfection group, *, the comparison between Mock group and NC group, *P* < 0.05 (NC, negative control)

Figure 2 shows that the expression level of miR-26b in both Mock group and NC group were 1.05 ± 0.05 and 1.04 ± 0.06, respectively (*P* > 0.05). The expression level in mimic group was 2.56 ± 0.15, which was higher than the Mock group and NC group (all *P* < 0.05). In miR-26b inhibitor group, miR-26b expression level was 0.23 ± 0.04, significantly lower than Mock and NC groups (all *P* < 0.05).

### Confirmation of target-gene of miR-26b by dual-luciferase reporter gene system

**Fig. 3:**
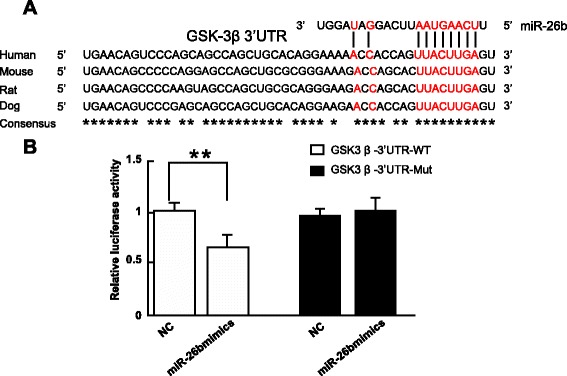
GSK-3β is the target of miR-26b. **a**: the comparison between miR-26b and 3′UTR of GSK-3β, the red part is complementary sites (core sequence, also can be regarded as miR-26b is act on the hypothetical gene loci of GSK-3β gene; GSK-3β 3′-UTR is highly conserved between species) **b**: Dual-luciferase assay for reporter gene analysis; detected in RAFLS cotransfected miR-26b mimics and GSK-3β 3′-UTR Wt/Mu plasmid; miR-26b inhibited activity of luciferase containing Wt 3′-UTR, *P < 0.05, while Mut plasmid activity of luciferase showed no change. NC, negative control

Using bioinformatics Target Scan software (http://www.targetscan.org), the potential target-genes of miR-26b were analyzed. The 3′-UTR of GSK-3β was highly conserved between different species and contained the binding site for mir-26b (Fig. 3a). As shown in Fig. 3b, recombinant plasmids containing Wt- miR-26b/GSK-3β and Mut- miR-26b/GSK-3β were constructed and cotransfected with miR-26b mimics. The results of the dual luciferase activity assays indicated that miR-26b mimics did not influence luciferase activity in Mut- miR-26b/GSK-3β, but sharply decrease luciferase activity when the 3′-UTR contained Wt- miR-26b/GSK-3β (all *P* < 0.05).

### Expression of nt4, Wnt5ɑ, GSK-3β, CyclinD1, phosphorylationSer9-GSK-3βand β-catenin

**Fig. 4:**
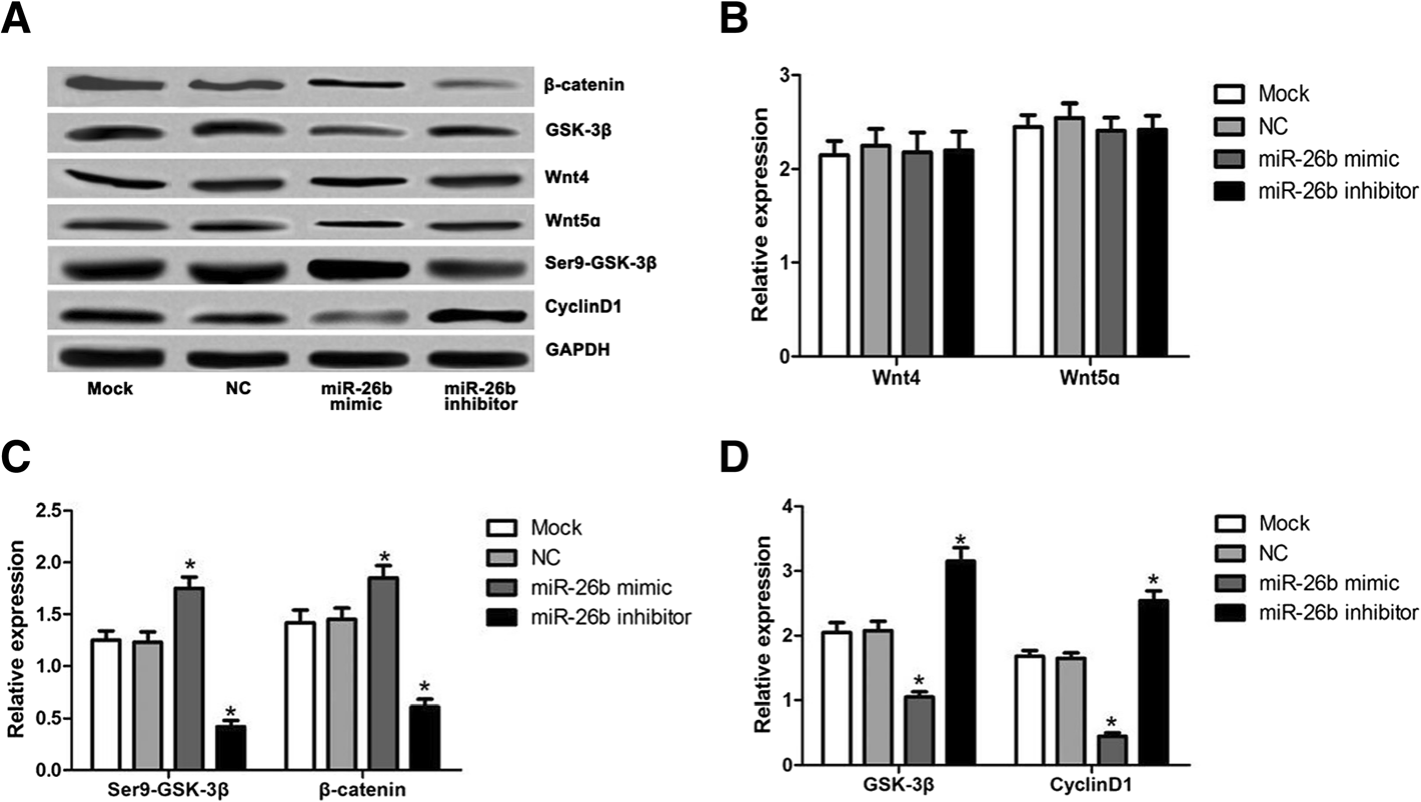
Expression of Wnt4, Wnt5ɑ, Ser9-GSK-3β, CyclinD1 and β-catenin, *, the comparison between Mock group and NC group, P < 0.05. **a**, Western blot of Wnt4, Wnt5ɑ, Ser9-GSK-3β, CyclinD1 and β-catenin expression; **b**, relative expression of Wnt4 and Wnt5ɑ in the 4 experimental groups; **c**, relative expression of Ser9-GSK-3β andβ-catenin in the 4 groups; **d**: relative expression of GSK-3β and CyclinD1in the 4 groups (4 groups: Mock, NC, miR-26b mimic and miR-26b inhibitor group)**.** NC, negative control

As shown in Fig. 4, Wnt4 and Wnt5 expression level in each group showed no significant differences (*P* > 0.05). By contrast, GSK-3β and CyclinD1 expression levels in miR-26b mimic group were lower than the Mock group and NC group (all *P* < 0.05). Conversely, the expression level of GSK-3β and CyclinD1 in the miR-26b inhibitor group was higher than the Mock and NC group (*P* < 0.05). In the miR-26b mimic group, expression levels of Ser9-GSK-3β and β-catenin were higher than Mock and NC groups (all *P* < 0.05), while in the miR-26b inhibitor group, their levels were significantly lower (all *P* < 0.05). The expression of GSK-3β, CyclinD1, Ser9-GSK-3β and β-catenin were not different statistically (all *P* > 0.05).

### TNF-ɑ, IL- 1β and IL-6 levels

**Fig. 5:**
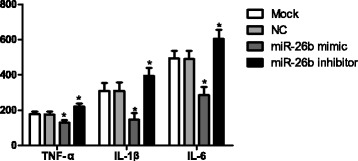
The ELISA results of TNF-ɑ,L- 1βand IL-6 levels, *, the comparison between Mock group and NC group, *P* < 0.05. NC, negative control

TNF-α, IL- 1β and IL-6 levels were quantified by ELISA (Fig. 5). TNF-α, IL- 1β and IL-6 levels showed no statistical differences between the Mock and NC groups (all *P* > 0.05). However, in the miR-26b mimic group, these cytokine levels were significantly lower than the Mock and NC groups (all *P* < 0.05). On the other hand, in the miR-26b inhibitor group, the cytokines levels were higher than in the Mock and NC groups (all *P* < 0.05).

### Proliferation capacity of RAFLS

**Fig. 6:**
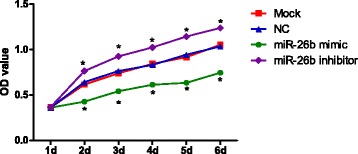
The MTT assay results for cell proliferation, *, comparison between Mock group and NC group, *P* < 0.05. NC, negative control

The RAFLS cell proliferation was measured by MTT method, as shown in Fig. 6. We observed no differences in cell proliferation capacity between the Mock group and NC group (*P* > 0.05). In miR-26b mimic group, the RAFLS cell proliferation capacity was significantly lower than the Mock and NC groups (all *P* < 0.05). In the miR-26b inhibitor group, the proliferation capacity of RAFLS was significant higher than Mock and NC groups (all *P* < 0.05).

### Effects of MiR-26b on RAFLS apoptosis

**Fig. 7:**
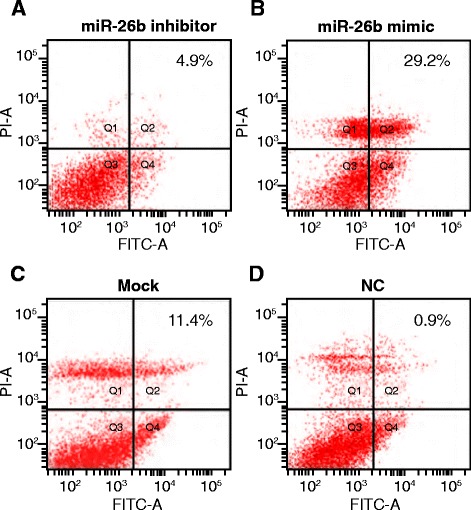
Effects of miR-26b on RAFLS apoptosis (Q1:necrotic cells; Q2:apoptotic cells; Q3:living cells; Q4:early apoptotic cells. Apoptosis rate of RAFLS in miR-26b inhibitor group is lower than Mock and NC group, while the rate in miR-26b mimic group is higher than Mock and NC group; apoptosis rates between Mock and NC group showed no significant differences). NC, negative control

As shown in Fig. 7, RAFLS apoptosis rates between the Mock and NC groups were not significantly different (*P* > 0.05). However, in the miR-26b mimic group, RAFLS apoptosis was markedly higher than observed in the Mock and NC groups (all *P* < 0.05). In the miR-26b inhibitor group, RAFLS apoptosis was significant lower than the Mock and NC groups (all *P* < 0.05).

## Results and discussion 

RA is a chronic systemic disease characterized by inflammatory synovitis [33]. RA pathology is influenced by several risk factors such as environmental factors, activated cellular pathways, viral infections, genetic factors, hormonal influences and psychological state [34]. RA is associated with significant comorbidities, articular damage, disability and increased mortality [35]. MiRNAs are a new class of small no-coding single-stranded RNAs 13–21 nucleotides in length and function as post-transcriptional regulators of gene expression by complementary binding to target mRNAs [36, 37]. MiRNAs are excellent candidates as molecular biomarkers for diagnosis and prognosis in several human pathological conditions [38, 39]. Shang et al. showed that miRNAs also influence the disease course in severe joint diseases, including osteoarthritis and RA, through regulating diverse cellular processes such as cell proliferation, differentiation, signal transduction, immune response and apoptosis [40].

In our study, miR-26b elevated the expression of β-catenin and CyclinD1 by lowering GSK-3β expression, which in-turn activated Wnt/GSK-3β/β-catenin pathway, inhibited RAFLS apoptosis and led to increased secretion of TNF-α,IL-1β and IL-6. Thus, miR-26b plays a central role in pathways controlling inflammation in reumatoid arthritis. Wnt is a secreted protein important in cell proliferation, differentiation, cell morphology, cell adhesion, cell motility and development [41]. In addition, β–catenin has a dual function in regulating gene transcription and in cell–cell adhesion [42]. As a component of the cadherin protein complex, β–catenin is an intracellular signal transducer of cell polarity and also functions in the Wnt signaling pathway [43]. Jong Hui Suh et al. demonstrated that miR-26a, which has the same core sequence with miR-26b, is indeed a functional gene product and is capable of binding to GSK-3 β and directly regulating its expression [44]. GSK-3β is a regulator of β-catenin levels and Wnt-induced intracellular signaling sequesters β-catenin from GSK-3β and promotes its nuclear accumulation, allowing β-catenin to complex with T-cell factor/lymphocyte enhancer binding factor (TCF/LEF) to activate transcription of target genes [45]. GSK-3β, functions at the upstream step of Wnt/β-catenin signaling pathway and is one of the most important negative regulatory component in the cell to control β-catenin function [46]. GSK-3β directly regulates β-catenin availability in the nucleus, depending on the intensity of the Wnt signaling pathway, by phosphorylating β-catenin in the cytosol and targeting it for ubiquitin-dependent proteolysis [47, 48]. GSK-3β was later found to regulate key signaling events relating to multiple aspects of cellular function, including protein synthesis, cytoskeletal integrity and gene expression [49]. In this study, transfection of miR-26b led to a significant increase in total GSK-3β and a decrease in the inhibitory phosphorylation on Ser9-GSK-3β, strongly influencing Wnt/GSK-3β/β-catenin pathway. Therefore, we believe that miR-26b is specific to this pathway and has a prominent role in inhibiting RA synovial inflammation. Consistent with this interpretation, Wnt-4 and Wnt-5α showed no significant differences in our study. Wnt-5α alters cell morphology by reducing cell adhesion and members of this family activate the non-canonical Wnt pathway.

Our study revealed that transfection of miR-26b significantly inhibited NF-κB activity, as judged by the sharply decreased levels of tumor necrosis factor (TNF)-α, IL-1β and IL-6. Although the exact mechanism of this effect need to be further investigated, we believe that miR-26b is intimately involved in RA progression and miR-26b based strategies have the potential to be highly effective against synovium inflammation in RA, consistent with earlier observations by Turner-Brannen et al. [50]. Our study is also supported by Tomoyuki Nakasa et al., who presented convincing results that NF-κB activity is critical for the initiation and maintenance of chronic inflammation in RA synovial tissue [15].

It is interesting to note a significant decrease in RASF proliferation after miR-26b transfection. Compared with healthy individuals, hyperplasia of synovial cells is induced by excessive cell proliferation or/and defects in apoptosis, leading to synovial lining thickness of up to 10–15 cell layers [51, 52]. Synovial hyperplasia can also promote the synovium attachment to the adjoining cartilage and bone, which causes joint dysfunction [53]. Clinically, arthroscopic synovectomy is very affective in patients with high synovial hyperplasia in local joints, suggesting that hyperplasia of synovial cells plays an important role in RA pathology and joint function [54].

## Conclusions

Based on our results, we propose that the proliferation of synovial fibroblast is a significant event in RA and inhibition of hyperplasia may be an effective treatment for RA. MiR-26b may be useful to down-regulate SF hyperplasia and inhibit synovium inflammation. In summary, miR-26b inhibits RASF proliferation and reduces secretion of inflammatory cytokines, including TNF-α,IL-1β and IL-6, via inhibiting Wnt/GSK-3β/β-catenin pathway through regulating GSK-3β level.
